# Management of symptomatic cholelithiasis: a systematic review

**DOI:** 10.1186/s13643-022-02135-8

**Published:** 2022-12-12

**Authors:** Rivfka Shenoy, Patrick Kirkland, Joseph E. Hadaya, M. Wynn Tranfield, Michael DeVirgilio, Marcia M. Russell, Melinda Maggard-Gibbons

**Affiliations:** 1 Department of Surgery, UCLA David Geffen School of Medicine, Los Angeles, CA USA; 2 Veterans Health Administration, Greater Los Angeles Healthcare System, Los Angeles, CA USA; 3 National Clinician Scholars Program, UCLA, Los Angeles, CA USA; 4 Department of Surgery, Los Angeles County Harbor-UCLA Medical Center, Los Angeles, CA USA; 5 Louise M. Darling Biomedical Library, UCLA Library, University of California, Los Angeles, CA USA; 6 Rand Corporation, Santa Monica, CA USA; 7 Olive View-UCLA Medical Center, Sylmar, CA USA

**Keywords:** Symptomatic cholelithiasis, biliary colic, treatment, management, cholecystectomy, UDCA

## Abstract

**Background:**

Symptomatic cholelithiasis is a common surgical disease and accounts for half of the over one million cholecystectomies performed in the USA annually. Despite its prevalence, only one prior systematic review has examined the evidence around treatment strategies and it contained a narrow scope. The goal of this systematic review was to analyze the clinical effectiveness of treatment options for symptomatic cholelithiasis, including surgery, non-surgical therapies, and ED pain management strategies.

**Methods:**

Literature search was performed from January 2000 through June 2020, and a narrative analysis was performed as studies were heterogeneous.

**Results:**

We identified 12 publications reporting on 10 trials (9 randomized controlled trials and 1 observational study) comparing treatment methods. The studies assessed surgery, observation, lithotripsy, ursodeoxycholic acid, electro-acupuncture, and pain-management strategies in the emergency department. Only one compared surgery to observation.

**Conclusion:**

This work presents the existing data and underscores the current gap in knowledge regarding treatment for patients with symptomatic cholelithiasis. We use these results to suggest how future trials may guide comparisons between the timing of surgery and watchful waiting to create a set of standardized guidelines. Providing appropriate and timely treatment for symptomatic cholelithiasis is important to streamline care for a costly and prevalent disease.

**Trial registration:**

PROSPERO Protocol Number: CRD42020153153

**Supplementary Information:**

The online version contains supplementary material available at 10.1186/s13643-022-02135-8.

## Introduction

Fifteen percent of Americans have gallstones and symptoms occur in up to 10% of patients within 5 years, which can progress to advanced disease such as acute cholecystitis, choledocholithiasis, or gallstone pancreatitis [1–6]. Gallstones lead to over one million ambulatory care visits each year, are a leading cause of hospital admissions, and result in one million cholecystectomies annually [7–9]. Symptomatic cholelithiasis, often referred to as biliary colic, accounts for half of these surgeries [2, 8, 9]. Despite being a common surgical problem, there is no consensus nor formal recommendations for eligibility criteria or optimal timing for surgery for symptomatic cholelithiasis.

However, surgery for symptomatic cholelithiasis may not always be warranted. The majority of patients with gallstone disease will not experience recurrent symptoms or disease progression [4, 5]. Patients may opt for observation alone, which may in part depend on how pain is managed in the emergency department (ED) [4, 5, 10]. Others may pursue non-surgical treatment options, including extracorporeal shock-wave lithotripsy or medical treatments such as ursodeoxycholic acid (UDCA), but success rates for such options are unclear [11]. While one prior systematic review focused on surgery as a treatment modality [1], none have comprehensively analyzed the evidence across the range of treatment options. The goal of this systematic review was to analyze the clinical effectiveness of treatment options for symptomatic cholelithiasis, including surgery, non-surgical therapies, and ED pain management strategies.

## Materials and methods

This systematic review is reported using PRISMA standards and the protocol for the larger review was registered in PROSPERO: CRD42020153153. One librarian developed a search strategy for comparing treatment methods for symptomatic cholelithiasis.

### Literature search

All searches included PubMed, Embase, Cochrane Trials and Cochrane Reviews from January 2000 to 29 June 2020, when the search was executed. The search strategy used a broad set of terms related to the treatment outcomes of cholelithiasis, gallbladder, and biliary tract disease (see Supplementary material 1 for complete search strategy). The search emphasized terms indicating length of stay, hospital readmission, and quality adjusted life years to ascertain post-intervention impacts. We excluded studies published prior to the year 2000 to capture contemporary treatment strategies.

### Study selection and data collection

**Fig. 1:**
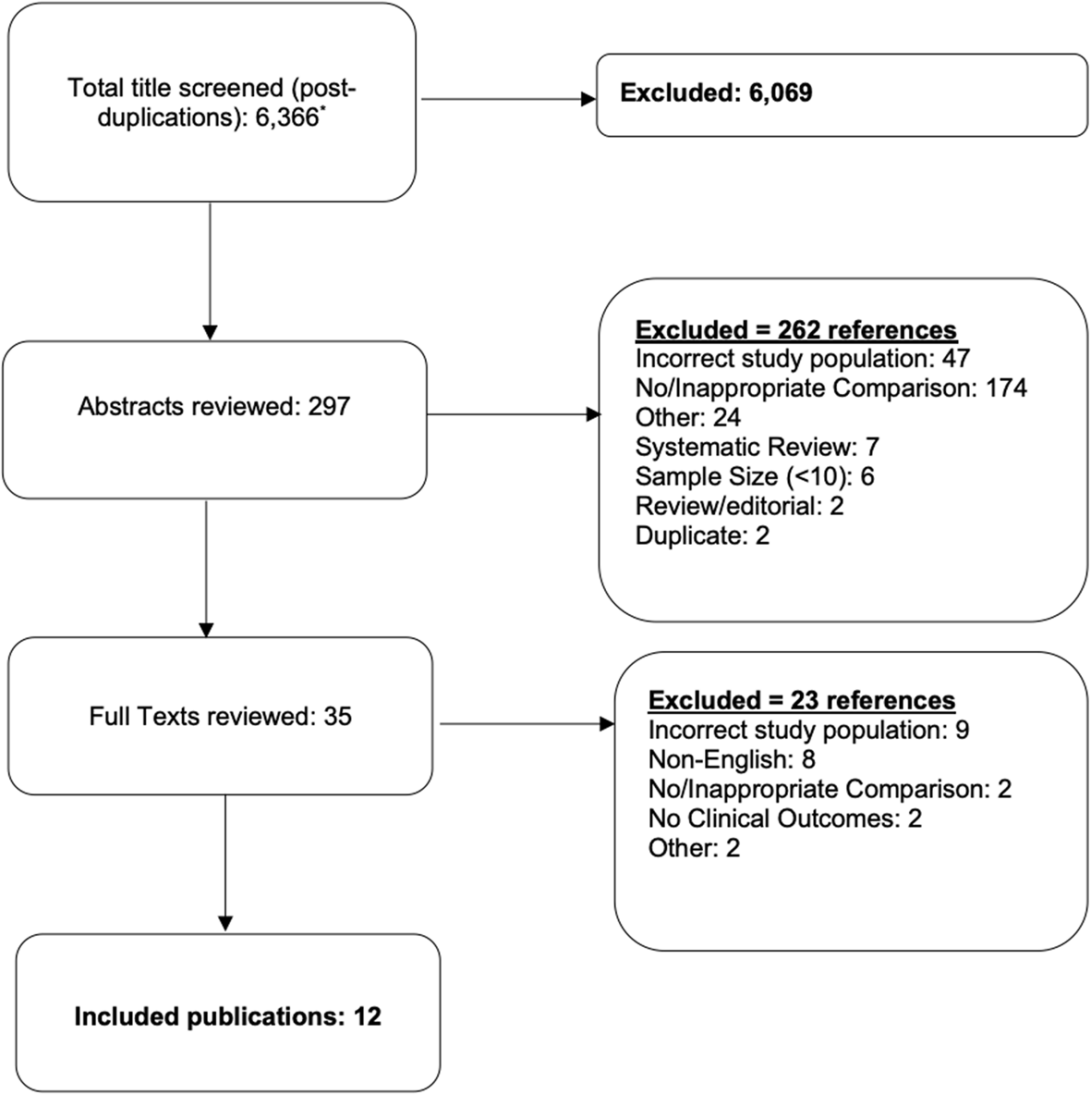
Literature flow. ^*^PubMed = 2575, Cochrane Trials 909, Cochrane Review 25, Embase 2838

All stages of title screen through data abstraction were completed by two independent team members and disagreements were reconciled through discussion. Studies that did not compare treatments were excluded. Studies were included if they assessed surgery (cholecystectomy), non-surgical therapies, or ED pain management strategies as one of the comparison arms. Studies were included if they had all of the following criteria: (1) studied adult patients with symptomatic cholelithiasis or included a sub-group with symptomatic cholelithiasis; (2) included one group of patients treated by observation or alternate treatment method; (3) had a comparison to patients treated with a different method; (4) measured intraoperative, perioperative, or postoperative outcomes. Randomized controlled trials (RCTs) and observational studies were included. We did not exclude studies based on follow-up time. Abstracts were included in the review (if there was no companion full article) and underwent the same quality assessment and duplication exclusion as full texts. Exclusion criteria are listed in our literature flow (Fig. 1).

Dual abstraction was performed including: study design, patient characteristics, sample size, intraoperative outcomes, postoperative outcomes, long-term functional outcomes, duration of follow-up, and data needed for the Cochrane Risk of Bias tool or Cochrane Risk of Bias In Non-randomized Studies—of Interventions (ROBINS-I) [12, 13]. Summary statistics (means, medians, or percentage as appropriate) describing differences between treatment groups were extracted.

### Risk of bias

RCTs were assessed for quality (risk of bias) with the Cochrane Risk of Bias tool [12]. We used the ROBINS-I [13] for observational studies. Each outcome was measured on consistency, directness, and precision with an overall certainty of evidence of high, moderate, low, or very-low.

### Statistical analysis

Due to the heterogeneity in clinical outcomes of both the RCTs and the observational studies, a meta-analysis was not performed, and data was synthesized narratively. Studies were grouped based on the types of treatments compared: surgery, (surgery versus observation, surgery versus lithotripsy, urgent versus elective surgery or surgical criteria comparisons), non-surgical therapies (UDCA versus placebo, UDCA versus UDCA with chenodeoxycholic acid, and electro-acupuncture versus observation), and pain management in the ED.

## Results

### Literature search

The search identified 6366 publications and 12 were included in our study (see Fig. 1 for literature flow and breakdown by database) [14–25]. These 12 articles reported on nine RCTs [14–17, 19–25] (several duplicates) and one observational study [18]. Table 1 shows the comparison arms and study characteristics for each study including follow-up time. For surgery comparative studies, seven publications reported on five trials (three reported different time-points and outcomes for one RCT) [14–20] Specifically, three publications reported on one trial that compared surgery versus observation [15, 16, 19], two compared timing of surgery [17, 18], one compared surgery to lithotripsy [14], and one compared methods to select patients for surgery [20]. Three publications compared non-surgical therapies [21, 22, 25]. Two compared UDCA to either placebo [22] or UDCA plus chenodeoxycholic acid [21], and one compared electro-acupuncture to observation [25]. Two publications compared types of ED pain medication [23, 24]. Supplementary material 2 displays the full-data extraction tables for all 12 studies.

**Table 1:** Characteristics of included studies by comparative arms: surgery, non-surgical, and ED pain management

Author, year	Comparison	Number of sites	Study design	Sample size	Follow-up time
Vetrhus, 2002 [16], Vetrhus 2004 [15], and Schmidt, 2011 [19]^a^	Surgery vs. observation	Multiple	Randomized	137	5 years [15, 16] 14 years [19]
Ahmed, 2000 [14]	Surgery vs. lithotripsy	Single	Randomized	144	5 years
Salman, 2005 [17]	Urgent vs. elective surgery	Single	Randomized	75	Not specified
Anwar, 2008 [18]^b^	Urgent vs. elective surgery	Single	Observational	96	Not specified
Van Dijk, 2019 [20]^c^	Surgeon discretion vs. pre-specified criteria	Multiple	Randomized	1067	1 year
Petroni, 2001 [21]	UDCA^d^ vs. UDCA + bile salts	Multiple	Randomized	158	2 years
Venneman, 2006 [22]	UDCA vs. placebo	Multiple	Randomized	177	Varied^e^
Wong, 2019 [25]	Electro-acupuncture vs. observation	n/a	Randomized	46	Not specified
Malesci, 2003 [23]	ED pain management^e^	Single	Randomized	14	48h
Antevil, 2004 [24]	ED pain management^f^	Single	Randomized	38	20 min

^a^Studies looked at same population, examined different outcomes at different timepoints

^b^Anwar, 2008 defined urgent cholecystectomy as early/same-day

^c^Surgeon discretion defined as operation based on standard care left to the discretion of the surgeon; restrictive strategy used the fulfillment of five pre-specified criteria as indication for operation

^d^*UDCA* Ursodeoxycholic acid

^e^Followed until they received surgery or for 12 months from start of treatment if refused surgery

^e^Hyoscine-N-butyl bromide vs. Loxiglumide

^f^Glycopyrrolate vs. placebo

### Study characteristics by comparison group

The seven publications (reporting on five trials) which included a surgical comparison arm had sample sizes ranging from 75 to 1067 patients. Two trials were single institution [14–16] and the other three were multi-institution (Table 1) [17, 18, 20]. Four out of five trials reported that groups were similar in regard to age and sex. Of these four, only one study demonstrated a statistical difference between groups [16] and the other three did not report statistical tests of comparisons [14, 16, 20]. The fifth trial (Anwar, et al.) which included a surgical comparison only reported age of the patients and did not comment on statistical significance [18]. Three out of five trials defined symptomatic cholelithiasis as abdominal pain with ultrasound signs of gallstones and without evidence of advanced biliary pathology (i.e., abnormal leukocytes, complicated cholelithiasis) [15–17, 19, 20]. One trial included patients with “symptomatic gallstones” without further specifying [14], and one included patients with cholelithiasis based on clinical findings from the chart [18]. Three out of five trials reported specific clinical disease characteristics including number of prior episodes, severity of prior episodes, prior hospitalizations and length of symptoms [14, 16, 20]. These trials did not report statistical tests of comparison, but stated that characteristics were similar between groups. These trials had follow-up times ranging from 1 [20] to 14 years [19]. Two trials did not specify their follow-up time (Table 1) [17, 18].

The two multi-institutional comparisons including UDCA had sample sizes of 158 [21] and 177 [22]. Both groups defined symptomatic cholelithiasis as abdominal pain lasting at least 30 min with gallstones, and without advanced biliary disease [21, 22]. The first study found no differences in age, sex, or weight between groups. This study reported a number of different baseline disease characteristics such as number of biliary colic episodes in the preceding year, pain localization, and pain medications needed in the preceding year. There were no significant differences in these characteristics between groups [22]. The other study reported age, sex, BMI, and stone characteristics, stating that groups were well-matched, but did not report statistical tests of comparison [21]. The follow-up times for these studies were 1 [22] and 2 years [21]. The study examining electro-acupuncture was an abstract only (unknown number of institutions), did not report differences in demographics between groups, and defined their cohort as those with “symptomatic gallstones” [25]. This study did not specify follow-up time.

The two studies looking at ED pain management were both single-institution and enrolled less than 50 patients [23, 24]. They found no differences in age, sex, or duration of pain between comparison arms. Both defined their cohort as patients with right upper quadrant abdominal pain with gallstones on ultrasound. One study specifically mentioned excluding patients with acute cholecystitis [23]. This study reported number of prior episodes and pain score at enrollment and identified no differences between groups [23]. The follow-up time for these studies were 48 h [23] and 20 min [24].

### Surgical comparisons: surgery versus observation

One RCT examined surgery versus observation and published three studies (Vetrhus, 2002; Vetrhus, 2004; Schmid, 2011) looking at different outcomes at different time-points [15, 16, 19]. Gallstone-related events including pain attacks and complications were not different between groups at 5 or 14 years (Table 2). Over half of the patients in the observation group received surgery (50.7%). Conversion rates and postoperative complications were slightly higher in the patients randomized to observation that ultimately underwent surgery (conversion rates: 11% versus 0; postoperative complications: 14% versus 5%, Table 3); however, they did not report whether this difference was statistically significant. Vetrhus, et al. (2004) examined quality of life (using the Psychological General Well Being index and Nottingham Health Profile Part II) and pain (pain score and visual analog pain scale) and found no differences between the surgery versus observation group [15].

**Table 2:** Outcomes of gallstone-related events and operative rate by surgical comparison

Author, year	Definition of gallstone-related events	Gallstone-related events		Operative rate	
		*Surgery*	*Observation*	*Surgery*	*Observation*
Vetrhus, 2002 [16] and 2004 [15] and Schmidt, 2011 [19]^*^	Complications of gallstones: acute pancreatitis, common bile duct stone(s), acute cholecystitis	5-year follow-up Pain-related admissions: 2 (1%) Complications: 1 (1%) 14-year follow-up Pain attacks: 8 (12%) Complications: 1 (1%)	5-year follow-up Pain-related admission: 12 (17%) Complications: 3 (4%) 14-year follow-up Pain attacks: 23 (33%) Complications: 3 (4%)	60/68 randomized (88%)	35/69 randomized (51%)
		*Elective* *surgery*	*Urgent/early* *surgery*	*Elective surgery*	*Urgent/early surgery*
Salman, 2005 [17]	“Complications during the waiting time”	9 (27.5%)	n/a	100%	
Anwar, 2008 [18]^b^	“serial presentations with symptoms of gallstones”	1.2 visits/person^c^ 0.3 visits/person^d^	n/a	100%	
		*Surgeon* *discretion*	*Restrictive* *strategy*	*Surgeon discretion*	*Restrictive strategy*
Van Dijk, 2019 [20] ^e^	“Gallstone complications”	38 (7%)	40 (8%)	404 (75%)	358 (68%)

^a^Studies looked at same population, examined different outcomes at different timepoints

^b^Anwar, 2008 defined urgent cholecystectomy as early/same-day

^c^In cohort that initially presented as an emergency

^d^In cohort that initially presented to outpatient

^e^Surgeon discretion defined as operation based on standard care left to the discretion of the surgeon; restrictive strategy used the fulfillment of five pre-specified criteria as indication for operation

**Table 3:** Operative outcomes for surgical comparisons

Author, year	Wait time mean ± SD		Conversion rate ***N***, (%)		Postoperative complications ***N***, (%)	
	*Surgery*	*Observation*	*Surgery*	*Observation*	*Surgery*	*Observation*
Vetrhus, 2002 [16], 2004 [15], and Schmidt, 2011 [19]^a^	5-year follow-up 3 [0–24] ^b^ months 14-year follow-up 3 [0–168] months^d^	5-year follow-up 27 [0–67] months 14-year follow-up 28 months^b^	0	4 (11%)	3 (5%)	5 (14%)
	*Elective surgery*	*Urgent/early surgery*	*Elective surgery*	*Urgent/early surgery*	*Elective surgery*	*Urgent/early* *surgery*
Salman, 2005 [17]	4.2 ± 1.4 months	14.2 ± 4.1 h	6 (17.2%)^c^	0	0^e^	0
Anwar, 2008 [18]^f^	114 days	3 days	0^e^	2 (2%)	7 (8%)^e^	0
	*Surgeon discretion*	*Restrictive strategy*	*Surgeon discretion*	*Restrictive strategy*	*Surgeon discretion*	*Restrictive strategy*
Van Dijk, 2019 [20]^g^	6 weeks [2, 10]^c,h^	6 weeks [3, 11]^h^	7 (2%)^e^	7 (2%)	88 (22%)^e^	74 (21%)

aStudies looked at same population, examined different outcomes at different timepoints

^b^No range reported

^c^*p* < 0.05

^d^Median [range]

^e^Not significantly different

^f^Anwar, 2008 defined urgent cholecystectomy as early/same-day

^g^Surgeon discretion defined as operation based on standard care left to the discretion of the surgeon; restrictive strategy used the fulfillment of five pre-specified criteria as indication for operation

^h^Median [IQR]

### Surgical comparisons: surgery versus lithotripsy

Ahmed, et al. compared lithotripsy to surgery in a 5-year follow-up study to examine long-term health gains. Open, elective cholecystectomy was compared to inpatient lithotripsy consisting of up to four treatment sessions on consecutive days with up to 3000 shocks per session. This study found that while both groups had experienced reductions in mean number of episodes of biliary pain and mean severity summary score, patients treated with surgery had larger decrease in both measures as compared to the group treated with lithotripsy [14]. For example, 81.8% (*N* 45) of patients who underwent cholecystectomy were pain-free at 5-year follow-up compared to 55.2% (*N* 48) of patients who were randomized to lithotripsy (*p* < 0.05).

### Surgical comparisons: elective vs. urgent

Two studies compared timing of surgery for symptomatic cholelithiasis [17, 18]. Salman et al. compared urgent (within 24 h from presentation) versus elective surgery, and Anwar, et al. compared early or same-day (defined as an operation on the next available list) versus elective surgery. The wait times for each arm are shown in Table 3. Over one quarter of patients waiting for elective surgery required gallstone-related visits (Table 2). Of those patients who underwent urgent or early surgery, none had gallstone-related events. Salman, et al. found a reduction in conversion rates during surgery for the elective group (17.2% versus 0%, *p* < 0.05); however Anwar, et al. found no differences in conversion rates between the early versus elective groups (2% versus 0%, *p* > 0.05). Neither study found a difference in postoperative complications between groups with Salman, et al showing no postoperative complications in any groups, and Anwar, et al. finding 8% complication rates in the elective group and zero complications in the early group.

### Surgical comparisons: criteria for surgical eligibility

Van Dijk, et al. examined methods of selecting patients for surgery. Standard care in the participating centers (surgeon-discretion) was compared to a method using fulfillment of pre-specified criteria for eligibility in which a patient had to fulfill all five criteria to be eligible for operation (restrictive). The five pre-specified criteria were (1) severe pain attacks, (2) pain lasting 15–30 min or longer, (3) pain located in epigastrium or right upper quadrant, (4) pain radiating to the back, and (5) a positive pain response to simple analgesics [20]. There was no difference in proportion of patients who were pain-free at 1 year (surgeon-discretion: 60% vs restrictive: 56%, *p* > 0.05), or gallstone-related events (Table 2) based on surgery selection method [20]. There were also no differences in conversion rates (2% in both groups, *p* > 0.05), postoperative complications (surgeon discretion 21% versus restrictive 21%, *p* > 0.05) or gallstone complications between groups (surgeon discretion 7% versus restrictive 8%, *p* > 0.05) [20].

### Non-surgical therapies

Two RCTs examined the use of UDCA. Petroni et al. compared UDCA alone with UDCA with chenodeoxycholic acid and found that both treatments reduced the frequency of biliary pain at three months and throughout the 2-year follow-up (UDCA alone 26% versus UDCA with chenodeoxycholic acid 21%, *p* < 0.05). Since this was a secondary end-point, they did not compare the difference in reduction between groups. They found no substantial difference in gallstone dissolution rate (primary end-point) between groups at 2 years (UDCA alone 28% versus UDCA with chenodeoxycholic acid 30%, *p* > 0.05) [21]. Venneman et al compared UDCA to placebo in patients waiting for surgery and found no difference in the proportion of patients that were colic-free or experienced complications between groups [22]. For example, 26% (*N* 23) of patients receiving UDCA were colic-free compared to 33% (*N* 29) in the placebo group (*p* > 0.05) (follow-up time varied, see Table 1).

One study (abstract-only) compared electro-acupuncture versus observation [25]. Patient reported outcomes were only reported secondarily and they found no differences between groups. Of note, their primary outcome was proportion of patients with clearance of gallstones (confirmed by ultrasonography), and there was no difference in clearance between groups. In the electro-acupuncture group, 9% (*N* 2) of patients had full clearance, compared to 4% (*N* 1) in the control group (*p* > 0.05).

### ED pain management strategies

Two trials compared medications for pain management in the ED for patients with symptomatic cholelithiasis. Antevil et al. examined intravenous glycopyrrolate versus placebo and demonstrated no difference in the median decrease in pain (between zero and 20 min) using the visual analog pain scale (3 [95% CI − 2–2]) versus 1 [95% CI − 3, 12]) [24]. Malesci et al. compared loxiglumide (CCK-1 receptor blocker) versus hyoscine-N-butyl bromide (anticholinergic) and found that the reduction in pain score as measured by visual analog scale was significantly greater with loxiglumide after 20 (88% vs 47%, *p* < 0.05) and 30 min (92% vs 49%, *p* < 0.05) [23]. This study also found that a second injection was needed in fewer patients treated with loxiglumide (14% vs 86%, *p* < 0.05) at 30 min.

### Risk of bias

The risk of bias for the RCTs which had a surgical arm was judged to be moderate (Supplementary material 3) [14–17, 19, 20]. Studies were deemed to have a moderate rating due to high risk of bias pertaining to blinding of participants, personnel across all studies and a high risk of bias in blinding of outcome assessment for most studies (one study had unknown risk of bias) [20]. The one observational study which had a surgical arm had a moderate risk of bias using the ROBINS-I tool due to non-random assignment of treatment arms [18].

The risk of bias for the RCTs comparing UDCA treatment was low was judged to be low with one study having low risk across all categories assessed [22], and the other having low or unknown risk across all categories [21]. The RCT comparing electro-acupuncture to observation was rated as high risk, with bias across most domains (Supplementary material 3) [25]. The RCTs comparing ED pain management strategies were low risk across all domains [23, 24].

## Discussion

This systematic review found 12 publications reporting on 10 trials (9 RCTs and 1 observational study) comparing treatment methods for symptomatic cholelithiasis. The studies assessed surgery, observation, lithotripsy, UDCA, electro-acupuncture and pain-management strategies in the ED. We identified only one trial that compared surgery to observation, one comparing surgery to lithotripsy, two comparing timing of surgery and one comparing methods to select patients for surgery. Non-surgical alternatives included two studies examining the use of UDCA (either comparing to placebo or in a combination therapy) and one examining the use of electro-acupuncture compared to observation. Two studies looked at options for pain management in the ED. Given this heterogeneity, making conclusions across studies was limited, and this review highlights challenges in studying treatments for a disease process that may present at varied stages of disease.

When interpreting the data, the time course of patients’ symptomatic cholelithiasis should be considered. Prior literature demonstrated that over half of patients with symptomatic cholelithiasis will not experience recurrence of symptoms after their first attack [25, 26]. Thus, the patient’s disease severity is critical. Of the 10 studies in our review, only 6 reported on symptoms or stone characteristics at randomization or presentation [13, 15, 19–22] and only two reported statistical tests comparing these factors [21, 22]. Patients were enrolled at all different stages of disease presentation with one study including those who had zero prior attacks along with those who had over five pain attacks a month [15]. The varied disease course of symptomatic cholelithiasis makes findings difficult to interpret when patients are studied at different presentations, and makes designing and performing RCTs difficult in this field. Perhaps, in order to guide clinicians when counseling patients with symptomatic cholelithiasis, future trials should stratify patients based on their disease presentation of symptomatic cholelithiasis (i.e., number of prior episodes, duration or severity of pain). Such trials may then consider interventions based on this stratification, for example randomizing patients to watchful waiting versus surgery early in their disease presentation, or to urgent versus elective surgery if they present after several attacks. Another consideration for patients with mild symptoms is to utilize a placebo arm. Such a study would randomize patients to laparoscopic cholecystectomy versus placebo procedure (no actual surgery), and examine whether those in the placebo arm continued to have symptoms. A similar study in orthopedic surgery demonstrated that surgical intervention in patients with osteoarthritis did not provide better outcomes than in those who underwent the placebo procedure [27]. This study design may be more interpretable than a watchful waiting versus surgery trial to delineate patients with symptomatic cholelithiasis who would benefit from gallbladder removal.”

Despite these challenges, two studies concluded that fewer complications were associated with early (within 24 h) or urgent cholecystectomy for symptomatic cholelithiasis as compared to elective surgery [16, 17]. This was based primarily on complications during the waiting period for patients receiving elective surgery, with both studies reporting mean surgery wait times of over three months. Prior literature showed that prolonged wait times for elective cholecystectomy can be associated with patient morbidity and increased hospital costs. One study found that while waiting for cholecystectomy, 14% of patients required an unplanned presentation to the hospital [28]. However, operating immediately for non-emergent disease processes is also not ideal since urgent procedures have higher morbidity and mortality than elective procedures [29]. Identifying and capitalizing on the optimal time to operate is not easy since both urgent surgery and long wait times are associated with complications. Implementing strategies to minimize surgical wait times while avoiding the need to operate urgently may prevent complications and alleviate the costly burden of this disease [7, 9]. A better understanding of which patients may be more likely to experience complications can guide prioritization to reduce recurrent ED visits while waiting for surgery. One study examined these factors by looking at age, sex, diagnosis, and comorbidities and found that only older age was associated with longer wait times for surgery [30]. However there were several characteristics missing, such as patient’s access to care, socioeconomic status, and information about the treating hospital. These characteristics may provide insight to identify vulnerable groups at higher risk for experiencing complications while waiting for surgery.

This systematic review has several limitations. Within our treatment grouping categories, there was heterogeneity between patient factors and clinical outcomes assessed. Some studies primarily examined clinical outcomes, while others focused on quality of life or health status. Studies that focused on clinical outcomes measured gallstone-related events in different ways, with some focusing on pain-related admissions and complications separately [15, 18], and others grouping all complications together [16, 17]. Additionally, we were unable to test for publication bias and cannot make any conclusions about its possible existence. Finally, overall quality of the studies was low to moderate, given unclear blinding mechanisms for RCTs and non-random assignment of treatment arms for the observational study. Despite these limitations, our work provides a current, comprehensive analysis of treatment strategies for symptomatic cholelithiasis.

Based on our findings, medical or alternate therapies for symptomatic cholelithiasis such as UDCA, lithotripsy, or electro-acupuncture as compared to surgery or watchful waiting have not been well studied. Studies comparing the timing of surgery or watchful waiting at particular points in patient’s disease process are warranted to determine optimal management and create a set of standardized guidelines to guide clinicians when counseling patients. Providing appropriate and timely treatment for symptomatic cholelithiasis is important to streamline care for a costly and prevalent disease.

## Supplementary Information

**Additional file 1: Supplementary material 1.** Search strategies.

**Additional file 2: Supplementary material 2.** Data extraction tables.

**Additional file 3: Supplementary material 3.** Risk of bias for randomized controlled trials and observational studies.

## Data Availability

The datasets generated and analyzed during the current study are available in Supplementary material 2. The articles used to generate these evidence tables are available in the PubMed, Embase, or Cochrane repository.
