# Supplementary material for: Management of symptomatic cholelithiasis: a systematic review
Source: Syst Rev. 2022 Dec 12;11:267. doi: 10.1186/s13643-022-02135-8 (PMC9743645; doi:10.1186/s13643-022-02135-8)
Supplement: Supplementary file 1 — Additional file 1: Supplementary material 1. Search strategies. [file 13643_2022_2135_MOESM1_ESM.docx]

**Supplementary Material 1. Search Strategies**

**PubMed Search:**

*Filters applied: 80 and over: 80+ years, Adult: 19+ years, Young Adult: 19-24 years, Adult: 19-44 years, Middle Aged + Aged: 45+ years, Middle Aged: 45-64 years, Aged: 65+ years, Human.*

((((((((((((((((("randomized clinical trial"[Other Term] OR "randomized clinical trials"[Other Term]) OR "randomised clinical trial"[Other Term]) OR "randomised clinical trials"[Other Term]) OR "randomized controlled trial"[Other Term]) OR "randomized controlled trials"[Other Term]) OR "randomised controlled trial"[Other Term]) OR "randomised controlled trials"[Other Term]) OR ((((((("randomized clinical trial"[Title/Abstract] OR "randomized clinical trials"[Title/Abstract]) OR "randomised clinical trial"[Title/Abstract]) OR "randomised clinical trials"[Title/Abstract]) OR "randomized controlled trial"[Title/Abstract]) OR "randomized controlled trials"[Title/Abstract]) OR "randomised controlled trial"[Title/Abstract]) OR "randomised controlled trials"[Title/Abstract])) AND (("Treatment Outcome"[MeSH Terms] OR ("treatment outcome/organization and administration"[MeSH Terms] OR "treatment outcome/statistics and numerical data"[MeSH Terms])) OR ("treatment outcome/organization and administration"[MeSH Major Topic] OR "treatment outcome/statistics and numerical data"[MeSH Major Topic]))) OR "Time Factors"[MeSH Terms]) OR "Time Factors"[MeSH Major Topic]) OR "quality-adjusted life years"[MeSH Terms]) OR "cost-benefit analysis"[MeSH Terms]) OR "Elective Surgical Procedures"[MeSH Terms]) OR "Patient Readmission"[MeSH Terms]) OR ((((((((((("Patient Readmission"[Title/Abstract] OR "Patient Readmission"[Text Word]) OR "Patient Readmission"[Other Term]) OR "cost benefit analysis"[Title/Abstract]) OR "cost benefit analysis"[Text Word]) OR "cost benefit analysis"[Other Term]) OR "Quality-Adjusted Life Years"[Title/Abstract]) OR "Quality-Adjusted Life Years"[Text Word]) OR "Quality-Adjusted Life Years"[Other Term]) OR "Treatment Outcome"[Title/Abstract]) OR "Treatment Outcome"[Text Word]) OR "Treatment Outcome"[Other Term])) AND ((((((((((((((((((((("gallston*"[Other Term] OR "gallston*"[Text Word]) OR "gallston*"[Title/Abstract]) OR "Gallstones"[MeSH Terms]) OR "gallstones/diagnosis"[MeSH Terms]) OR "gallstones/economics"[MeSH Terms]) OR "gallstones/organization and administration"[MeSH Terms]) OR "gallstones/statistics and numerical data"[MeSH Terms]) OR "gallstones/surgery"[MeSH Terms]) OR "gallstones/therapy"[MeSH Terms]) OR "Gallstones"[MeSH Major Topic]) OR "gallstones/diagnosis"[MeSH Major Topic]) OR "gallstones/economics"[MeSH Major Topic]) OR "gallstones/organization and administration"[MeSH Major Topic]) OR "gallstones/statistics and numerical data"[MeSH Major Topic]) OR "gallstones/surgery"[MeSH Major Topic]) OR "gallstones/therapy"[MeSH Major Topic]) OR (((((((((((("gallbladder*"[Other Term] OR "gallbladder*"[Text Word]) OR "gallbladder*"[Title/Abstract]) OR "Gallbladder Diseases"[MeSH Major Topic]) OR "gallbladder diseases/diagnosis"[MeSH Major Topic]) OR "gallbladder diseases/organization and administration"[MeSH Major Topic]) OR "gallbladder diseases/statistics and numerical data"[MeSH Major Topic]) OR "gallbladder diseases/surgery"[MeSH Major Topic]) OR "Gallbladder Diseases"[MeSH Terms]) OR "gallbladder diseases/diagnosis"[MeSH Terms]) OR "gallbladder diseases/organization and administration"[MeSH Terms]) OR "gallbladder diseases/statistics and numerical data"[MeSH Terms]) OR "gallbladder diseases/surgery"[MeSH Terms])) AND (((((((((((((((((((((((((((("Biliary Tract Diseases"[MeSH Terms] OR "biliary tract diseases/adverse effects"[MeSH Terms]) OR "biliary tract diseases/analysis"[MeSH Terms]) OR "biliary tract diseases/classification"[MeSH Terms]) OR "biliary tract diseases/complications"[MeSH Terms]) OR "biliary tract diseases/diagnosis"[MeSH Terms]) OR "biliary tract diseases/economics"[MeSH Terms]) OR "biliary tract diseases/organization and administration"[MeSH Terms]) OR "biliary tract diseases/prevention and control"[MeSH Terms]) OR "biliary tract diseases/rehabilitation"[MeSH Terms]) OR "biliary tract diseases/statistics and numerical data"[MeSH Terms]) OR "biliary tract diseases/surgery"[MeSH Terms]) OR "biliary tract diseases/therapy"[MeSH Terms]) OR "Biliary Tract Diseases"[MeSH Major Topic]) OR "biliary tract diseases/adverse effects"[MeSH Major Topic]) OR "biliary tract diseases/analysis"[MeSH Major Topic]) OR "biliary tract diseases/classification"[MeSH Major Topic]) OR "biliary tract diseases/complications"[MeSH Major Topic]) OR "biliary tract diseases/diagnosis"[MeSH Major Topic]) OR "biliary tract diseases/economics"[MeSH Major Topic]) OR "biliary tract diseases/organization and administration"[MeSH Major Topic]) OR "biliary tract diseases/prevention and control"[MeSH Major Topic]) OR "biliary tract diseases/rehabilitation"[MeSH Major Topic]) OR "biliary tract diseases/statistics and numerical data"[MeSH Major Topic]) OR "biliary tract diseases/surgery"[MeSH Major Topic]) OR "biliary tract diseases/therapy"[MeSH Major Topic]) OR "biliary tract"[Other Term]) OR "biliary tract"[Text Word]) OR "biliary tract"[Title/Abstract])) OR (((((((((((((((((("gallston*"[Other Term] OR "gallston*"[Text Word]) OR "gallston*"[Title/Abstract]) OR "Gallstones"[MeSH Terms]) OR "gallstones/diagnosis"[MeSH Terms]) OR "gallstones/economics"[MeSH Terms]) OR "gallstones/organization and administration"[MeSH Terms]) OR "gallstones/statistics and numerical data"[MeSH Terms]) OR "gallstones/surgery"[MeSH Terms]) OR "gallstones/therapy"[MeSH Terms]) OR "Gallstones"[MeSH Major Topic]) OR "gallstones/diagnosis"[MeSH Major Topic]) OR "gallstones/economics"[MeSH Major Topic]) OR "gallstones/organization and administration"[MeSH Major Topic]) OR "gallstones/statistics and numerical data"[MeSH Major Topic]) OR "gallstones/surgery"[MeSH Major Topic]) OR "gallstones/therapy"[MeSH Major Topic]) OR (((((((((((("gallbladder*"[Other Term] OR "gallbladder*"[Text Word]) OR "gallbladder*"[Title/Abstract]) OR "Gallbladder Diseases"[MeSH Major Topic]) OR "gallbladder diseases/diagnosis"[MeSH Major Topic]) OR "gallbladder diseases/organization and administration"[MeSH Major Topic]) OR "gallbladder diseases/statistics and numerical data"[MeSH Major Topic]) OR "gallbladder diseases/surgery"[MeSH Major Topic]) OR "Gallbladder Diseases"[MeSH Terms]) OR "gallbladder diseases/diagnosis"[MeSH Terms]) OR "gallbladder diseases/organization and administration"[MeSH Terms]) OR "gallbladder diseases/statistics and numerical data"[MeSH Terms]) OR "gallbladder diseases/surgery"[MeSH Terms])) AND (((((((((((((((((("Colic"[MeSH Terms] OR "colic/analysis"[MeSH Terms]) OR "colic/classification"[MeSH Terms]) OR "colic/diagnosis"[MeSH Terms]) OR "colic/economics"[MeSH Terms]) OR "colic/organization and administration"[MeSH Terms]) OR "colic/prevention and control"[MeSH Terms]) OR "colic/rehabilitation"[MeSH Terms]) OR "colic/statistics and numerical data"[MeSH Terms]) OR "colic/surgery"[MeSH Terms]) OR "Colic"[MeSH Major Topic]) OR "colic/analysis"[MeSH Major Topic]) OR "colic/diagnosis"[MeSH Major Topic]) OR "colic/economics"[MeSH Major Topic]) OR "colic/organization and administration"[MeSH Major Topic]) OR "colic/prevention and control"[MeSH Major Topic]) OR "colic/rehabilitation"[MeSH Major Topic]) OR "colic/statistics and numerical data"[MeSH Major Topic]) OR "colic/surgery"[MeSH Major Topic]))) OR (((((((((((((((((("gallston*"[Other Term] OR "gallston*"[Text Word]) OR "gallston*"[Title/Abstract]) OR "Gallstones"[MeSH Terms]) OR "gallstones/diagnosis"[MeSH Terms]) OR "gallstones/economics"[MeSH Terms]) OR "gallstones/organization and administration"[MeSH Terms]) OR "gallstones/statistics and numerical data"[MeSH Terms]) OR "gallstones/surgery"[MeSH Terms]) OR "gallstones/therapy"[MeSH Terms]) OR "Gallstones"[MeSH Major Topic]) OR "gallstones/diagnosis"[MeSH Major Topic]) OR "gallstones/economics"[MeSH Major Topic]) OR "gallstones/organization and administration"[MeSH Major Topic]) OR "gallstones/statistics and numerical data"[MeSH Major Topic]) OR "gallstones/surgery"[MeSH Major Topic]) OR "gallstones/therapy"[MeSH Major Topic]) OR (((((((((((("gallbladder*"[Other Term] OR "gallbladder*"[Text Word]) OR "gallbladder*"[Title/Abstract]) OR "Gallbladder Diseases"[MeSH Major Topic]) OR "gallbladder diseases/diagnosis"[MeSH Major Topic]) OR "gallbladder diseases/organization and administration"[MeSH Major Topic]) OR "gallbladder diseases/statistics and numerical data"[MeSH Major Topic]) OR "gallbladder diseases/surgery"[MeSH Major Topic]) OR "Gallbladder Diseases"[MeSH Terms]) OR "gallbladder diseases/diagnosis"[MeSH Terms]) OR "gallbladder diseases/organization and administration"[MeSH Terms]) OR "gallbladder diseases/statistics and numerical data"[MeSH Terms]) OR "gallbladder diseases/surgery"[MeSH Terms])) AND (((((((((((((("cholecystectomy/economics"[MeSH Terms] OR "cholecystectomy/organization and administration"[MeSH Terms]) OR "cholecystectomy/statistics and numerical data"[MeSH Terms]) OR "cholecystectomy/trends"[MeSH Terms]) OR "cholecystectomy/economics"[MeSH Major Topic]) OR "cholecystectomy/organization and administration"[MeSH Major Topic]) OR "cholecystectomy/statistics and numerical data"[MeSH Major Topic]) OR "cholecystectomy/trends"[MeSH Major Topic]) OR "cholecystectomy, laparoscopic/organization and administration"[MeSH Major Topic]) OR "cholecystectomy, laparoscopic/statistics and numerical data"[MeSH Major Topic]) OR "cholecystectomy, laparoscopic/organization and administration"[MeSH Terms]) OR "cholecystectomy, laparoscopic/statistics and numerical data"[MeSH Terms]) OR "cholecystectomy"[Other Term]) OR "cholecystectomy"[Text Word]) OR "cholecystectomy"[Title/Abstract]))) OR (((((((((((("Postcholecystectomy Syndrome"[MeSH Terms] OR "postcholecystectomy syndrome/diagnosis"[MeSH Terms]) OR "postcholecystectomy syndrome/prevention and control"[MeSH Terms]) OR "postcholecystectomy syndrome/statistics and numerical data"[MeSH Terms]) OR "postcholecystectomy syndrome/surgery"[MeSH Terms]) OR "Postcholecystectomy Syndrome"[MeSH Major Topic]) OR "postcholecystectomy syndrome/diagnosis"[MeSH Major Topic]) OR "postcholecystectomy syndrome/prevention and control"[MeSH Major Topic]) OR "postcholecystectomy syndrome/statistics and numerical data"[MeSH Major Topic]) OR "postcholecystectomy syndrome/surgery"[MeSH Major Topic]) OR "postcholecystectom*"[Title/Abstract]) OR "postcholecystectom*"[Text Word]) OR "postcholecystectom*"[Other Term]))) NOT ((((((((("gallstone pancreatitis"[Title/Abstract] OR "gallstone pancreatitis"[Other Term]) OR "gallbladder cancer"[Title/Abstract]) OR "gallbladder cancer"[Other Term]) OR "biliary cancer"[Title/Abstract]) OR "biliary cancer"[Other Term]) OR "Postcholecystectomy Syndrome"[Title/Abstract]) OR "Postcholecystectomy Syndrome"[Other Term]) OR "acalculus cholecystitis"[Other Term]) OR "acalculus cholecystitis"[Title/Abstract])

**Embase Search: Jan 2000 to June 2020**

('cholelithiasis'/exp OR 'gallstone'/exp OR 'biliary tract surgery'/exp OR 'gallbladder disease'/exp OR 'biliary tract disease'/exp OR 'colic'/exp OR 'cholecystectomy'/exp OR 'laparoscopic cholecystectomy'/exp OR 'postcholecystectomy syndrome'/exp) AND ('gallstone' OR 'gallbladder disease' OR 'biliary tract disease' OR 'colic' OR 'postcholecystectomy syndrome') NOT ('gallstone pancreatitis'/exp/mj OR 'gallbladder cancer'/exp/mj OR 'bile duct cancer'/exp/mj OR 'postcholecystectomy syndrome'/exp/mj OR 'acalculous cholecystitis'/exp/mj OR 'gallstone pancreatitis'/exp OR 'gallbladder cancer'/exp OR 'bile duct cancer'/exp OR 'postcholecystectomy syndrome'/exp OR 'acalculous cholecystitis'/exp) AND ('treatment outcome'/exp OR 'time factor'/exp OR 'quality adjusted life year'/exp OR 'cost benefit analysis'/exp OR 'elective surgery'/exp OR 'hospital readmission'/exp) AND ('human'/de OR 'randomized controlled trial'/de OR 'randomized controlled trial topic'/de) AND ([adult]/lim OR [aged]/lim OR [middle aged]/lim OR [very elderly]/lim OR [young adult]/lim)

**Cochrane Reviews: Jan 2000 to June 2020**

1. "cholelithiasis" OR "gallstone*" OR "biliary tract surgery" OR "gallbladder disease*" OR "biliary tract disease*" OR "cholecystectomy" OR "laparoscopic cholecystectomy" OR "elective laparoscopic cholecystectomy" or "postcholecystectomy syndrome" or "symptomatic gallstone disease" or "gallstone disease*" or "symptomatic cholelithiasis"
2. "gallstone pancreatitis" OR "gallbladder cancer" OR "bile duct cancer" OR "acalculous cholecystitis"
3. "'treatment outcome" OR "time factor" OR "quality adjusted life year" OR "cost benefit analysis" OR "elective surgery" OR "hospital readmission" or "emergency medical services" or "length of stay" or "outcome*"
4. #1 not #2
5. #4 and #3

**Cochrane Trials: Jan 2000 to June 2020**

## ("cholelithiasis" OR "gallstone*" OR "biliary tract surgery" OR "gallbladder disease*" OR "biliary tract disease*" OR "cholecystectomy" OR "laparoscopic cholecystectomy" OR "elective laparoscopic cholecystectomy" or "postcholecystectomy syndrome" or "gallstone disease*" or "biliary pain" or "symptomatic gallstone disease" or "gallbladder disease" OR "biliary tract disease" OR "postcholecystectomy syndrome" or "gallstone disease*" or "symptomatic gallstone disease"):ti,ab,kw NOT ("gallstone pancreatitis" OR "gallbladder cancer" OR "bile duct cancer" OR "acalculous cholecystitis"):ti,ab,kw AND ("treatment outcome" OR "time factor" OR "quality adjusted life year" OR "cost benefit analysis" OR "elective surgery" OR "hospital readmission" or "emergency medical services" or "length of stay")
