# Supplementary material for: Management of symptomatic cholelithiasis: a systematic review
Source: Syst Rev. 2022 Dec 12;11:267. doi: 10.1186/s13643-022-02135-8 (PMC9743645; doi:10.1186/s13643-022-02135-8)
Supplement: Supplementary file 2 — Additional file 2: Supplementary material 2. Data extraction tables. [file 13643_2022_2135_MOESM2_ESM.docx]

**Supplementary Material 2: Data Extraction Tables**

Patient Characteristics:

| Author  Year  Study Design US (y/n)  Propensity-Matching | #Institutions/Surgeons | Total | Elective (interval) Cholecystectomy | Urgent Cholecystectomy | Cholecystectomy (other timing- specify timing in column I) | | Lithotripsy | UDCA (specify dosage/timing in column L) | | Observation | Other 1 (specify in column N) | | Other 2 (specify in column P) | |
| --- | --- | --- | --- | --- | --- | --- | --- | --- | --- | --- | --- | --- | --- | --- |
| Vetrhus 2002 RCT N N | 3 hospitals/3 surgeons | N: 137 | N: 68 Women's age: 52 (20-77) Men's age: 52 (27-74) Men: 19.1% (13) Prior hospitalization for biliary colic: 20.6% (14) First episode: 5.9% (4) |  |  |  |  |  |  | N: 69 Women's age: 48 (22-75) Men's age: 60 (39-79) Men: 17.4% (12) Prior hospitalization for biliary colic: 23.2% (16) First episode: 8.7% (6) |  |  |  |  |
| Vetrhus 2004 RCT N N | 3 hospitals/3 surgeons | N: 137 | N: 68 Women's age: 52 (20-77) Men's age: 52 (27-74) Men: 19.1% (13) |  |  |  |  |  |  | N: 69 Women's age: 48 (22-75) Men's age: 60 (39-79) Men: 17.4% (12) |  |  |  |  |
| Schmidt 2011 RCT N N | 3/unknown | N:137 Age: 49.7 (range 20-79) Male: 18.2% |  |  | N:68 |  |  |  |  | N:69 |  |  |  |  |
| Salman 2005 Randomized N N | 1/unknown | N:75 | N:35 45.3 (2.6) ASA 1: 24 (68.6%) ASA 2: 9 (25.8%) ASA 3: 2 (5.7%) Male: 13 (37.1%) BMI 23.66 (2.06) | N:28 Age: 40.2 (9.4) ASA 1: 19 (67.85%)  ASA 2: 8 (28.57%) ASA 3: 1 (3.57%) BMI: 24.21 (2.16) |  |  |  |  |  |  |  |  |  |  |
| Anwar 2008  Observational, retrospectiveN  N | 1 institution/na | N: 9634 acute cholecystitis (data not extracted). | N: 90Age: 52yrPresented to accident/emergency: 13Presented to outpatient: 77 |  | N: 6Age: 55yr | Early/Same admission: operation on the next available listMean days until operation 3 |  |  |  |  |  |  |  |  |
| van Dijk 2019 RCT N N | 24/unknown | N:1067 |  |  |  |  |  |  |  |  | N: 537 (665, 112 excluded) Age (median, IQR): 49 (39-58) Male: 150 (28%) ASA 2: 86 (16%) BMI (median, IQR): 27.5 (24.6-31.2) | Usual care: cholecystectomy left to discretion of surgeon | N: 530 (661, 121 excluded) Age: 48.0 (37.0-59.0) Male: 131 (25%) ASA 2: 81 (15%) BMI: 27.5 (24.5-30.9) | Restrictive strategy: 5 prespecified criteria (severe pain attacks, pain lasting >15-30 min, epigastric or right upper quadrant, radiation to back, positive pain response to analgesics)  If not fulfilled consideration of alternative treatment/diagnosis pursued; followed up at 3, 6, 9, 12mo |
| Ahmed 2000 RCT (5yr FU) N N | 1 institution/na | N: 144 | N: 57 Age (median, range): 55 (43-66.5) Male: 28% (16) Median Bulk of Stones (mm3): 3770 (1717-4864) Median Diameter of Largest Stone (mm): 14.2 (8.1-20.4)  Biliary pain in last 3mo: 84% (48) Median number of episodes: 3 (1-13) Median severity of last episode (VAS): 67 (51-82) Stone number: 1: 35% (20) 2-3: 15% () 4-9: 15% (9) >=10: 33% (19) Number of prior episodes: 3, 1-13 (median, IQR) |  |  |  | N: 48 Age (median, range): 54 (44-64) Male: 37% (18) Median Bulk of Stones (mm3): 1560 (688-4378) Median Diameter of Largest Stone (mm): 11.4 (7.9-16.4)  Biliary pain in last 3mo: 85% (41) Median number of episodes: 3 (1-7) Median severity of last episode (VAS): 71 (39-85) Stone number: 1: 27% (13) 2-3: 25% (12) 4-9: 23% (11) >=10: 25% (11) Number of prior episodes: 3, 1-7 (median, IQR) |  |  |  | N: 39 Age (median, range): 53 (43-65) Male: 18% (7) Median Bulk of Stones (mm3): 1709 (584-5291) Median Diameter of Largest Stone (mm): 12.1 (7.0-21.4)  Biliary pain in last 3mo: 82% (32) Median number of episodes: 9 (1-25) Median severity of last episode (VAS): 49 (23-75) Stones/Patient 1: 44% (17) 2-3: 18% (7) 4-9: 10% (4) 10 or more: 28% (11) Median number of episodes: 9 (1-25) | Crossover Group (lithotripsy to cholecystectomy) |  |  |
| Petroni 2000 RCT N N | 6 centers/non-operative | N: 158 |  |  |  |  |  | N: 79 Age: 46.9 (SEM: 1.7) Men: 29.1% (23) BMI: 24.4 (SEM: 0.4) Stone no single: 25.3% (20) Stone no multiple 72.2% (57) Stone size (mm): 8.4 (SEM: 0.5)  Biliary pain in last 3mo: 59% | UDCA 10mg/kg, single bedtime dose |  | N: 79 Age: 49.8 (SEM 1.6) Men: 29.1% (23) BMI: 24.0 (SEM 0.4) Stone no single: 22.8% (18) Stone no multiple 74.7% (59) Stone size (mm): 8.9 (SEM: 0.5)  Biliary pain in last 3mo: 55% | UDCA + chenodeoxycholic acid tryhydrate magnesium salt (5+5mg/kg) administered as a single bedtime dose |  |  |
| Venneman 2006 RCT N N | 3 hospitals/na | N:177 |  |  |  |  |  | N: 89 Age (mean, SEM): 47 (1) Male: 21% Weight (kg, mean, SEM): 80 (2) Number of colic episodes in preceding year (mean, SEM): 14 (2) Smallest stone diameter (mm): 6 (1) Patients with low number of colic (<3): 16% | UDCA 750mg daily preoperative | N: 88 Age (mean, SEM): 45 (1) Male: 28% Weight (kg, mean, SEM): 84 (2) Number of colic episodes in preceding year (mean, SEM): 12 (2) Smallest stone diameter mm: 8 (1) Patients with low number of colic (<3): 20% | Ursofalk-Placebo 750mg daily preoperative | Venneman 2006 RCT N N | 3 hospitals/na | N:177 |
| Malesci 2003 RCT N N | 1/na | N: 14 |  |  |  |  |  |  |  |  | N: 7 Age (mean and SEM): 54.3 (4.0) Male: 43% (3) #Prior episodes: 3.0 (1.5) Pain score at enrollment: 7.7 (0.5) | hyoscine-N-butyl bromide (20mg), one dose. Second dose given 30min after first treatment if persistent pain | N: 7 Age (mean and SEM): 48.0 (4.7) Male: 29% (2) #Prior episodes: 3.7 (1.5) Pain score at enrollment: 6.3 (0.7) | Loxiglumide (50mg), one dose. Second dose given 30min after first treatment if persistent pain |
| Antevil  2004 RCT Y N | 1 institution | N: 38 |  |  |  |  |  |  |  |  | N: 21 Age: 34.4 Male: 42%  Mean temperature (farenheight): 97.5 Mean iniital pulse: 79 Mean WBC count: 11.3  Mean pain, time 0: 51 Diabetes (%): 0%  Previous abdominal surgery (%): 14  Patients with gallstones: 13 | Glycopyrrolate, 0.2mg IV administered, repeated for up to a total of 3 doses at 5-min intervals prn  Rescue analgesia provided after 20min | N: 17  Age: 34.9  Male: 31%  Mean temperature (farenheight): 98.1 Mean initial pulse: 75 Mean WBC count: 10.9 Mean pain, time 0: 63 Diabetes (%): 6 Previous abodminal surgery (%): 18 Patients with gallstones: 9 | Placebo: 0.2mg IV administered, repeated for up to a total of 3 doses at 5-min intervals prn.  Rescue analgesia provided after 20min |
| Wong  2019 RCT N N Abstract only | na/na | N: 46 |  |  |  |  |  |  |  | N: 24 Stone size: ≤ 8mm | N: 22 Stone size: ≤ 8mm | Electro-acupuncture: offered 20 sessions |  |  |

Operative Outcomes

| Author  Year  Study Design US (y/n)  Propensity-Matching | #Institutions/Surgeons | Elective (interval) Cholecystectomy | Urgent Cholecystectomy | Cholecystectomy (other timing) | Lithotripsy | UDCA | Observation | Other 1 | Other 2 |
| --- | --- | --- | --- | --- | --- | --- | --- | --- | --- |
| Vetrhus 2002 RCT N N | 3 hospitals/3 surgeons | Operation rate: 88.2% (60) Laparoscopic: 70% (45) Open: 30% (15) Converted to open: 0  Major complications: 5% (3) Intra-abdominal infection/bile leakage: 3.3% (2, major) Wound infection/dehiscence : 1.7% (1, major) Common bile duct stone/stenosis/pancreatitis: 0 (major) Minor complications: 10% (6) Reoperation: 1 (1.7%) |  |  |  |  | Operation rate: 50.7% (35) Laparoscopic: 82.9% (29) Open: 5.7% (2) Converted to open: 4 (11.4%) Major complications: 14.3% (5) Intra-abdominal infection/bile leakage: 8.6% (3, major) Wound infection/dehiscence : 2.9% (1, major) Common bile duct stone/stenosis/pancreatitis: 2.9% (1, major) Minor complications: 0 (0%) Reoperation: 1 (2.9%) |  |  |
| Vetrhus 2004 RCT N N | 3 hospitals/3 surgeons | Median time to Operation: 3mo (0-24) Operation Rate: 88.2% (60) Laparoscopic: 75% (45) Open: 25% (15) Major Complication: 3 (5%) Deaths: 4 (5.9%) Reoperation (bile leak): 1.7% (1) |  |  |  |  | Median Time to Operation: 27mo (0-67) Operation Rate: 50.7% (35) Laparoscopic: 82.9% (29) Open: 17.1% (6) Major Complication: 5 (14.3%) Deaths: 4 (5.8%) Reoperation (bile leak): 2.9% (1) |  |  |
| Schmidt 2011 RCT N N | 3/unknown |  |  | Wait Time Until Operation: 3 months (range 0-168) Operation Rate: 60 (88.2%) All events: 10 (29%) |  |  | Wait Time Until Operation: 28 months Operation Rate: 35 (50.7%) Patients with postoperative events: 8  Post-op complication number: 4  All events: 12 (20%) |  |  |
| Salman 2005 Randomized N N | 1/unknown |  |  | Wait Time Until Operation: 3 months (range 0-168) Operation Rate: 60 (88.2%) All events: 10 (29%) |  |  | Wait Time Until Operation: 28 months Operation Rate: 35 (50.7%) Patients with postoperative events: 8  Post-op complication number: 4  All events: 12 (20%) |  |  |
| Anwar 2008  Observational, retrospectiveN  N | 1 institution/na |  |  | Wait Time Until Operation: 3 months (range 0-168) Operation Rate: 60 (88.2%) All events: 10 (29%) |  |  | Wait Time Until Operation: 28 months Operation Rate: 35 (50.7%) Patients with postoperative events: 8  Post-op complication number: 4  All events: 12 (20%) |  |  |
| van Dijk 2019 RCT N N | 24/unknown |  |  |  |  |  |  | Wait Time (weeks, IQR): 6 (2.25-11.0) Operation Rate: 404 (75%) Surgical Complications: 88 (22%) Conversion to open: 7 (2%) | Wait Time (weeks, IQR): 6 (3-10) Operation Rate: 68% Surgical Complications: 74 (21%) Conversion to open: 7 (2%) |
| Ahmed 2000 RCT (5yr FU) N N | 1 institution/na |  | | | | | | | |
| Petroni 2000 RCT N N | 6 centers/non-operative |  |  |  |  |  |  |  |  |
| Venneman 2006 RCT N N | 3 hospitals/na |  |  |  |  |  |  |  |  |
| Malesci 2003 RCT N N | 1/na |  |  |  |  |  |  |  |  |
| Antevil  2004 RCT Y N | 1 institution |  |  |  |  |  |  |  |  |
| Wong  2019 RCT N N Abstract only | na/na |  |  |  |  |  |  |  |  |

Other Measured Outcomes

| Author  Year  Study Design US (y/n)  Propensity-Matching | #Institutions/Surgeons | Elective (interval) Cholecystectomy | Urgent Cholecystectomy | Cholecystectomy (other timing) | Lithotripsy | UDCA | Observation | Other 1 | Other 2 |
| --- | --- | --- | --- | --- | --- | --- | --- | --- | --- |
| Vetrhus 2002 RCT N N | 3 hospitals/3 surgeons | Admission due to pain: 2.9% (2- one was s/p surgery) Acute pancreatitis: 1.5% (1- patient did receive surgery) CBD Stone: 0  Acute cholecystitis: 0 |  |  |  |  | Admission due to pain: 21.7% (15, 6 in obs and 9 in surg) Acute pancreatitis: 0%  CBD stone: 2.9% (2) Acute chole: 1.4% (1) |  |  |
| Vetrhus 2004 RCT N N | 3 hospitals/3 surgeons | Gallstone-related events: 3 (4%) PGWB (baseline): 93.7 PGWB (6mo): 103.3 PGWB (12mo): 102.3 PGWB (60mo): 101.6 NHP (baseline): 2.0 NHP (6mo): 1.1 NHP (12mo): 1.0 NHP (60mo): 1.9 Pain Score (Baseline): 6.3 Pain Score (6mo): 2.2 Pain Score (12mo): 2.3 Pain Score (60mo): 2.0 VAPS (baseline): 47.2 VAPS (6mo): 9.4 VAPS (12mo): 7.0 VAPS (60mo): 5.5 |  |  |  |  | Gallstone-related events: 15 (22%)  PGWB (baseline): 95.2 PGWB (6mo): 99.9 PGWB (12mo): 101.8 PGWB (60mo): 104.1 NHP (baseline): 1.8 NHP (6mo): 1.6 NHP (12mo): 1.6 NHP (60mo): 1.3 Pain Score (baseline): 6.7 Pain Score (6mo): 4.1 Pain Score (12mo): 3.7 Pain Score (60mo): 2.4 VAPS (baseline): 48.1 VAPS (6mo): 14.7 VAPS (12mo): 15.7 VAPS (60mo): 11.5 |  |  |
| Schmidt 2011 RCT N N |  |  | Death at follow up: 11 (16.2%) Pain attacks post-op: 8 (13%) |  |  | Death at follow up: 8 (11.6%) Pain attacks post-op: 2 (6%) |  |  |  |
| Salman 2005 Randomized N N | 1/unknown | Complication rate during waiting period: 9 (25.7%) ED visits: 13 (among 9 patients) | Complication rate during waiting period: 0 (0%) |  |  |  |  |  |  |
| Anwar 2008  Observational, retrospectiveN  N | 1 institution/na | Further symptomatic presentations pre-operatively for those presenting as accident/emergency: 11 patients (out of 13), 15 visits (1.2 visits per patient in subgroup) Further symptomatic presentations pre-operatively for those presenting as outpatient: 19 patients out of 77), 23 visits (0.3 visits per patient in subgroup) |  | Further symptomatic presentations pre-operatively: 0 patients, 0 visits |  |  |  |  |  |
| van Dijk 2019 RCT N N |  |  |  |  |  |  | Gallstone Complications: 38 (7%) Pain-free at 12 months after cholecystectomy: 256 (63%) Pain-free at 12 months (conservative): 65 (49%) Patient reported satisfaction (median, IQR, 10-point scale): 8.4 (8-9) Time to pain-free irrespective of intervention: 7.29mo | Gallstone complications: 40 (8%) Pain-free at 12 months after cholecystectomy: 228 (64%) Pain-free at 12 months (conservative): 70 (41%) Patient reported satisfaction (median, IQR, 10-point scale): 8.4 (8-9.1) Time to pain-free, irrespective of intervention: 7.87mo |  |
| Ahmed 2000 RCT (5yr FU) N N | 1 institution/na | Pain-Free at 5yr follow-up: 81.8% (45)  Reduction in Pain (VAS): 44.8 (37 to 53) Reduction in Pain (McGill): 20.1 (16 to 24) NHP- Energy: 11.0 (23 to 120) NHP- Pain: 11.1 (4 to 18) NHP- Emotional Reaction: 8.1 (4 to 13) NHP- Sleep: 8.7 (2 to 16) NHP- Social Isolation: 1.0 (-2 to 5) NHP: Physical Mobility:- 0.4 (-6 to 4) |  |  | Pain-Free at 5 year Follow-up: 35 (74.5%) Reduction in Pain (VAS): 43.3 (32 to 54) Reduction in Pain (McGill): 18.6 (14 to 24) NHP- Energy: 16.6 (7 to 26) NHP- Pain: 2.1 (-4 to 9) NHP- Emotional Reaction: 8.0 (3 to 13) NHP- Sleep: 1.4 (-6 to 9) NHP- Social Isolation: 1.3 (-3 to 6) NHP: Physical Mobility:- -3.0 (-7 to -1) |  |  | Pain-Free at 5-year Follow-Up: 13 (35.1%) Reduction in Pain (VAS): 4.2 (-56 to 14) Reduction in Pain (McGill): 3.1 (-3 to 9) NHP- Energy: 16.2 (2 to 31) NHP- Pain: 11.7 (2 to 21) NHP- Emotional Reaction: 8.7 (2 to 16) NHP- Sleep:0.5 (-9 to 10) NHP- Social Isolation: -0.1 (-5 to 5) NHP: Physical Mobility: -1.3 (-7 to 4) |  |
| Petroni 2000 RCT N N | 6 centers/non-operative |  |  |  |  | Reduction in biliary pain frequency after 3mo: 59% to 26% Side-effects: 4 patients  Mean gallstone dissolution rate (6mo): 47% Mean gallstone dissolution rate: 59% (12mo) Complete dissolution (24mo): 28%  Patients w/ small stones (<= 5mm), complete dissolution 6mo: 46% Acquired gallstone calcification: 1 patient |  | Reduction in biliary pain frequency after 3mo: 55% to 21% Side effects: 6 patients  Mean gallstone dissolution rate (6mo): 44% Mean gallstone dissolution rate: 53% (12mo) Complete dissolution (24mo): 30%  Patients w/ small stones (<= 5mm), complete dissolution 6mo: 47% Acquired gallstone calcification: 2 patients |  |
| Venneman 2006 RCT N N | 3 hospitals/na |  |  |  |  | Reduction in biliary pain frequency after 3mo: 59% to 26% Side-effects: 4 patients  Mean gallstone dissolution rate (6mo): 47% Mean gallstone dissolution rate: 59% (12mo) Complete dissolution (24mo): 28%  Patients w/ small stones (<= 5mm), complete dissolution 6mo: 46% Acquired gallstone calcification: 1 patient |  | Reduction in biliary pain frequency after 3mo: 55% to 21% Side effects: 6 patients  Mean gallstone dissolution rate (6mo): 44% Mean gallstone dissolution rate: 53% (12mo) Complete dissolution (24mo): 30%  Patients w/ small stones (<= 5mm), complete dissolution 6mo: 47% Acquired gallstone calcification: 2 patients |  |
| Malesci 2003 RCT N N | 1/na |  |  |  |  | Reduction in biliary pain frequency after 3mo: 59% to 26% Side-effects: 4 patients  Mean gallstone dissolution rate (6mo): 47% Mean gallstone dissolution rate: 59% (12mo) Complete dissolution (24mo): 28%  Patients w/ small stones (<= 5mm), complete dissolution 6mo: 46% Acquired gallstone calcification: 1 patient |  | Reduction in biliary pain frequency after 3mo: 55% to 21% Side effects: 6 patients  Mean gallstone dissolution rate (6mo): 44% Mean gallstone dissolution rate: 53% (12mo) Complete dissolution (24mo): 30%  Patients w/ small stones (<= 5mm), complete dissolution 6mo: 47% Acquired gallstone calcification: 2 patients |  |
| Antevil  2004 RCT Y N | 1 institution |  |  |  |  |  |  | Median decrease in pain on visual analog scale, time 0-20min in all patients (95% CI): 3 (-2 to +17) Median decrease in pain on visual analog scale time 0-20min in paients with gallstones (95% CI): 3 (-2 to +20) | Median decrease in pain on visual analog scale, time 0-20min in all patients (95% CI): 8 (-2 to +20) Median decrease in pain on visual analog scale time 0-20min in paients with gallstones (95% CI): 1 (-3 to +12) |
| Wong  2019 RCT N N Abstract only | na/na |  |  |  |  |  | Total gallstone clearance: 1 (4.2%)  Partial gallstone clearance: 2 (8.3%) | Total gallstone clearance: 2 (9.1%)  Partial gallstone clearance: 4 (18.2%) |  |
