# Supplementary material for: Management of symptomatic cholelithiasis: a systematic review
Source: Syst Rev. 2022 Dec 12;11:267. doi: 10.1186/s13643-022-02135-8 (PMC9743645; doi:10.1186/s13643-022-02135-8)
Supplement: Supplementary file 3 — Additional file 3: Supplementary material 3. Risk of bias for randomized controlled trials and observational studies. [file 13643_2022_2135_MOESM3_ESM.docx]

**Supplementary Material 3**: Risk of Bias for Randomized Controlled Trials and Observational Studies

| Author, year | Random sequence generation | Allocation concealment | Blinding of participants and personnel | Blinding of outcome assessment | Incomplete outcome data | Selective reporting | Other sources of bias |
| --- | --- | --- | --- | --- | --- | --- | --- |
| Vetrhus, 2002 | ○ | ○ | ● | ● | ○ | ○ | n/a |
| Vetrhus, 2004 | ○ | ○ | ● | ● | ○ | ○ | n/a |
| Schmidt, 2011 | ○ | ○ | ● | ● | ○ | ○ | n/a |
| Ahmed, 2000 | ⯋ | ⯋ | ● | ● | ○^1^ | ○ | n/a |
| Salman, 2005 | ○ | ● | ● | ● | ⯋ | ○ | ○^2^ |
| Van Dijk, 2019 | ○ | ○ | ● | ⯋ | ○ | ○ | ○^3^ |
| Petroni, 2001 | ○ | ○ | ⯋ | ⯋ | ⯋ | ○ | n/a |
| Venneman, 2006 | ○ | ○ | ○ | ○^4^ | ○ | ○ | n/a |
| Wong, 2019 | ●^5^ | ●^5^ | ● | ● | ⯋ | ○ | n/a |
| Malesci, 2003 | ○ | ○ | ○ | ○ | ○ | ○ | n/a |
| Antevil, 2004 | ○ | ○ | ○ | ○ | ○ | ○ | n/a |

○ = low risk of bias ● = risk of bias ⯋ = unknown

^1^80% had data available for 5-year follow-up

^2^Acute cholecystitis (during waiting period, intraoperative, or on pathology) excluded after randomization

^3^Significant proportion of patients did not receive selected treatment (147/530) in restrictive strategy compared to usual care (12/537)

^4^ Details of blinding of outcome assessment not presented in article or clinicaltrials.gov, but reports that outcomes assessed by study physicians who were blinded

^5^This study was defined as a pragmatic RCT in clinicaltrials.gov and did not explain the protocol used for treatment decisions.

| **Author, year** | **Confounding** | **Selection bias** | **Bias in measurement classification of interventions** | **Bias due to deviations from intended interventions** | **Bias due to missing data** | **Bias in measurement of outcomes** | **Bias in selection of the reported result** | **Other source of bias** |
| --- | --- | --- | --- | --- | --- | --- | --- | --- |
| Anwar, 2008 | Serious: retrospective so unknown if treatment based on confounding clinical factors | Serious: retrospective so treatment arm based on unknown clinical factors, exclusion of 25% of patients | Low | Low | No information | Low | Low | n/a |

Options: low, moderate, serious, unknown
